# Primary abdominal ectopic pregnancy: a case report

**DOI:** 10.4076/1757-1626-2-8485

**Published:** 2009-08-07

**Authors:** Recep Yildizhan, Ali Kolusari, Fulya Adali, Ertan Adali, Mertihan Kurdoglu, Cagdas Ozgokce, Numan Cim

**Affiliations:** 1 Department of Obstetrics and Gynecology, School of Medicine, Yuzuncu Yil UniversityVanTurkey; 2 Department of Radiology, Women and Child HospitalVanTurkey

## Abstract

**Introduction:**

We present a case of a 13-week abdominal pregnancy evaluated with ultrasound and magnetic resonance imaging.

**Case presentation:**

A 34-year-old woman, (gravida 2, para 1) suffering from lower abdominal pain and slight vaginal bleeding was transferred to our hospital. A transabdominal ultrasound and magnetic resonance imaging were performed. The diagnosis of primary abdominal pregnancy was confirmed according to Studdiford’s criteria. A laparatomy was carried out. The placenta was attached to the mesentery of sigmoid colon and to the left abdominal sidewall. The placenta was dissected away completely and safely. No postoperative complications were observed.

**Conclusion:**

Ultrasound examination is the usual diagnostic procedure of choice. In addition magnetic resonance imaging can be useful to show the localization of the placenta preoperatively.

## Introduction

Abdominal pregnancy, with a diagnosis of one per 10000 births, is an extremely rare and serious form of extrauterine gestation [1]. Abdominal pregnancies account for almost 1% of ectopic pregnancies [2]. It has reported incidence of one in 2200 to one in 10,200 of all pregnancies [3]. The gestational sac is implanted outside the uterus, ovaries, and fallopian tubes. The maternal mortality rate can be as high as 20% [3]. This is primarily because of the risk of massive hemorrhage from partial or total placental separation. The placenta can be attached to the uterine wall, bowel, mesentery, liver, spleen, bladder and ligaments. It can be detach at any time during pregnancy leading to torrential blood loss [4]. Accurate localization of the placenta pre-operatively could minimize blood loss during surgery by avoiding incision into the placenta [5]. It is thought that abdominal pregnancy is more common in developing countries, probably because of the high frequency of pelvic inflammatory disease in these areas [6]. Abdominal pregnancy is classified as primary or secondary. The diagnosis of primary abdominal pregnancy was confirmed according to Studdiford’s criteria [7]. In these criteria, the diagnosis of primary abdominal pregnancy is based on the following anatomic conditions: 1) normal tubes and ovaries, 2) absence of an uteroplacental fistula, and 3) attachment exclusively to a peritoneal surface early enough in gestation to eliminate the likelihood of secondary implantation. The placenta sits on the intra-abdominal organs generally the bowel or mesentery, or the peritoneum, and has sufficient blood supply. Sonography is considered the front-line diagnostic imaging method, with magnetic resonance imaging (MRI) serving as an adjunct in cases when sonography is equivocal and in cases when the delineation of anatomic relationships may alter the surgical approach [8]. We report the management of a primary abdominal pregnancy at 13 weeks.

## Case presentation

**Figure 1.**
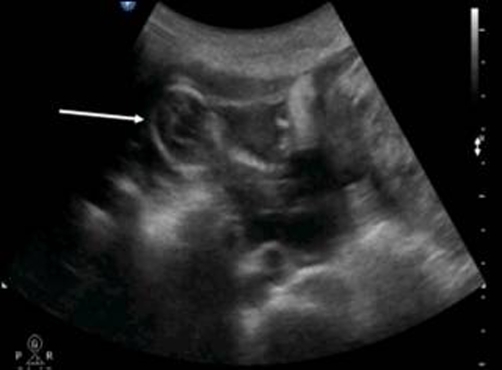
Pelvic ultrasound scanning. Diffuse free intraperitoneal fluid was seen around the fetus and small bowel loops.

**Figure 2.**
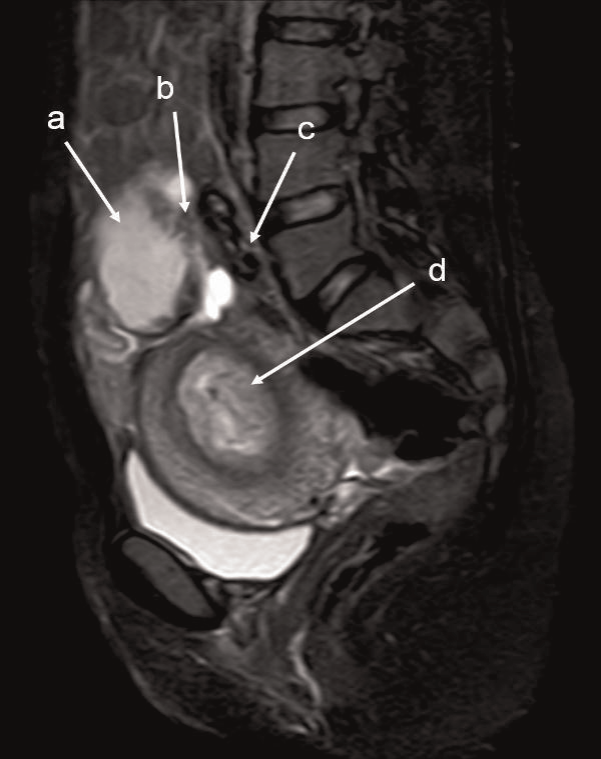
T2W SPAIR sagittal MRI of lower abdomen demonstrating the placental invasion. Placenta **(a)**, invasion area **(b)**, sigmoid colon **(c)**, uterine cavity **(d)**.

The patient was a 34-year-old Turkish woman, gravida 2 para 1 with a normal vaginal delivery 15 years previously. Although she had not used any contraceptive method afterwards, she had not become pregnant. She was transferred to our hospital from her local clinic at the gestation stage of 13 weeks because of pain in the lower abdomen and slight vaginal bleeding. She did not know when her last menstrual period had been, due to irregular periods. At admission, she presented with a history of abdominal distention together with steadily increasing abdominal and back pain, weakness, lack of appetite, and restlessness with minimal vaginal bleeding. She denied a history of pelvic inflammatory disease, sexually transmitted disease, surgical operations, or allergies. Blood pressure and pulse rate were normal. Laboratory parameters were normal, with a hemoglobin concentration of 10.0 g/dl and hematocrit of 29.1%. Transvaginal ultrasonographic scanning revealed an empty uterus with an endometrium 15 mm thick. A transabdominal ultrasound (Figure 1) examination demonstrated an amount of free peritoneal fluid and the nonviable fetus at 13 weeks without a sac; the placenta measured 58 × 65 × 67 mm. Abdominal-Pelvic MRI (Philips Intera 1.5T, Philips Medical Systems, Andover, MA) in coronal, axial, and sagittal planes was performed especially for localization of the placenta before she underwent surgery. A non-contrast SPAIR sagittal T2-weighted MRI strongly suggested placental invasion of the sigmoid colon (Figure 2).

**Figure 3.**
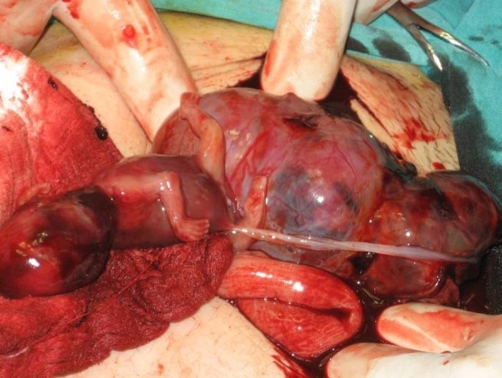
Fetus, placenta and bowels.

Under general anesthesia, a median laparotomy was performed and a moderate amount of intra-abdominal serohemorrhagic fluid was evident. The placenta was attached tightly to the mesentery of sigmoid colon and was loosely adhered to the left abdominal sidewall (Figure 3). The fetus was localized at the right of the abdomen and was related to the placenta by a chord. The placenta was dissected away completely and safely from the mesentery of sigmoid colon and the left abdominal sidewall. Left salpingectomy for unilateral hydrosalpinx was conducted. Both ovaries were conserved. After closure of the abdominal wall, dilatation and curettage were also performed but no trophoblastic tissue was found in the uterine cavity. As a management protocol in our department, we perform uterine curettage in all patients with ectopic pregnancy gently at the end of the operation, not only for the differential diagnosis of ectopic pregnancy, but also to help in reducing present or possible postoperative vaginal bleeding.

The patient was awakened, extubated, and sent to the room. The patient was discharged on post-operative day five with the standard of care at our hospital.

## Discussion

In the present case, we were able to demonstrate primary abdominal pregnancy according to Studdiford’s criteria with the use of transvaginal and transabdominal ultrasound examination and MRI. In our case, both fallopian tubes and ovaries were intact. With regard to the second criterion, we did not observe any uteroplacental fistulae in our case. Since abdominal pregnancy at less than 20 weeks of gestation is considered early [9], our case can be regarded as early, and so we dismissed the possibility of secondary implantation.

The recent use of progesterone-only pills and intrauterine devices with a history of surgery, pelvic inflammatory disease, sexually transmitted disease, and allergy increases the risk of ectopic pregnancy. Our patient had not been using any contraception, and did not report a history of the other risk factors.

The clinical presentation of an abdominal pregnancy can differ from that of a tubal pregnancy. Although there may be great variability in symptoms, severe lower abdominal pain is one of the most consistent findings [10]. In a study of 12 patients reported by Hallatt and Grove [11], vaginal bleeding occurred in six patients.

Ultrasound examination is the usual diagnostic procedure of choice, but the findings are sometimes questionable. They are dependent on the examiner’s experience and the quality of the ultrasound. Transvaginal ultrasound is superior to transabdominal ultrasound in the evaluation of ectopic pregnancy since it allows a better view of the adnexa and uterine cavity. MRI provided additional information for patients who needed precise diagnosing. After the diagnosis of abdominal pregnancy became definitive, it was essential to determine the localization of the placenta. Meanwhile, MRI may help in surgical planning by evaluating the extent of mesenteric and uterine involvement [12]. Non-contrast MRI using T_2_-weighted imaging is a sensitive, specific, and accurate method for evaluating ectopic pregnancy [13], and we used it in our case.

Removal of the placental tissue is less difficult in early pregnancy as it is likely to be smaller and less vascular. Laparoscopic removal of more advanced abdominal ectopic pregnancies, where the placenta is larger and more invasive, is different [14]. Laparoscopic treatment must be considered for early abdominal pregnancy [15].

Complete removal of the placenta should be done only when the blood supply can be identified and careful ligation performed [11]. If the placenta is not removed completely, it has been estimated that the remnant can remain functional for approximately 50 days after the operation, and total regression of placental function is usually complete within 4 months [16].

In conclusion, ultrasound scanning plus MRI can be useful to demonstrate the anatomic relationship between the placenta and invasion area in order to be prepared preoperatively for the possible massive blood loss.

## Abbreviations

MRI: Magnetic Resonance Imaging
SPAIR: Spectral Presaturation Attenuated by Inversion Recovery
